# Correction: Spectroscopic and microscopic examination of teeth exposed to green tea at different temperatures

**DOI:** 10.1371/journal.pone.0338964

**Published:** 2025-12-12

**Authors:** The PLOS One Staff

## Notice of Republication

This article was republished on December 2, 2025, to remove a Supporting Information file that was incorrectly included in the originally published article and to correct an error in the author list: William C. Cho was displayed as William Cho. The publisher apologizes for the errors. Please download this article again to view the correct version. The originally published, uncorrected article and the republished, corrected articles are provided here for reference.

## Supporting information

S1 File Originally published, uncorrected article. (PDF)

S2 File Republished, corrected article. (PDF)
