# Supplementary material for: Spectroscopic and microscopic examination of teeth exposed to green tea at different temperatures
Source: PLoS One. 2020 Dec 30;15(12):e0244542. doi: 10.1371/journal.pone.0244542 (PMC7773275; doi:10.1371/journal.pone.0244542)
Supplement: S1 File — (DOCX) [file pone.0244542.s005.docx]

**Spectroscopic and microscopic examination of teeth exposed to green tea at different temperatures**

Sinai H.C. Manno^1,2,3†^, Francis A.M. Manno^1,3†^, Li Tian^1,2^, Muhammad S. Khan^3^, Irfan Ahmed^3,4^, Yuanchao Liu^3^, Vincent W.T. Li^1^, Shisan Xu^1^, Fangjing Xie^1^, Tak Fu Hung^5^, Victor Ma^6^, William Cho^6^, Beatriz Aldape^7^, Shuk Han Cheng^1,8,^ †, Condon Lau^1^†

^1^Department of Biomedical Sciences, City University of Hong Kong, Hong Kong SAR, China

^2^State Key Laboratory of Marine Pollution (SKLMP), City University of Hong Kong, Hong Kong SAR, China

^3^Department of Physics, City University of Hong Kong, Hong Kong SAR, China

^4^Department of Electrical Engineering, Sukkur IBA University, Sukkur 65200, Sindh, Pakistan

^5^Department of Materials Science and Engineering, City University of Hong Kong, Hong Kong, China SAR

^6^Department of Clinical Oncology, Queen Elizabeth Hospital, Hong Kong

^7^División de Estudios de Posgrado e Investigación, Facultad de Odontología, Portal de la Universidad Nacional Autónoma de México, México, D.F., México.

^8^Jockey Club College of Veterinary Medicine and Life Sciences, City University of Hong Kong, Hong Kong SAR, China

**†Corresponding Authors**:

Condon Lau

Department of Physics

City University of Hong Kong

Hong Kong SAR, China

Email: [condon.lau@cityu.edu.hk](mailto:condon.lau@cityu.edu.hk)

Shuk Han Cheng

Department of Biomedical Sciences

Jockey Club College of Veterinary Medicine of Life Sciences

City University of Hong Kong

Hong Kong SAR, China.

Email: [bhcheng@cityu.edu.hk](mailto:bhcheng@cityu.edu.hk)

**Author Contributions:** designed research S.H.C.M., F.A.M.M., C.L.; performed research S.H.C.M., F.A.M.M., T.L., I.A., M.S.K., Y.L., S.X., F.X., T.F.H.; analyzed data S.H.C.M., F.A.M.M., V.W.L., B.A.B.; financial contributions: V.M., W.C., S.H.C., C.L.; wrote the paper S.H.C.M., F.A.M.M., C.L.

^†^Contributed equally.

**Key words**: elemental content, enamel surface, roughness, green tea, erosion, demineralization, staining.

**EXPERIMENTAL METHODS**

***Tea preparation***

For the *in vitro* experiment, teabags were dissolved in hot water. For the hot-tea group, the initial temperature of the water (87˚C) was measured with a thermometer. As the temperature dropped to 65˚C, teabags and jaws were immersed. For the *in vitro* experiment cold-tea group, the solution was allowed to cool to room temperature (RT) and then the jaws were immersed and maintained in the solution at RT. The samples were immersed into 50 ml of green tea solution in either cold or hot temperature. For *in vivo* experiments, tea leaves were put in hot water, allowed to cool to RT, and then placed in water bottles for rat consumption.

***In vivo and in vitro models***

Rats were deprived of water for 12 hours prior to embarking on the in vivo tea experiment to encourage drinking tea. Bottles (500 mL) were filled with green tea solution and allowed to cool (cold group) to RT. Rats were provided tea ad libitum. After seven days, jaws were extracted as described above. The samples were collected and grouped as in vivo tea and in vivo water (2 groups, where n=3 jaw per group, n=24 teeth per group, 48 teeth total). For in vitro analysis, jaws were placed in a 50 ml tube and divided into four groups: cold-water, hot-water, cold-tea, and hot-tea (4 groups, where n=3 bilateral jaw per group, n=24 teeth per group, 96 teeth total).

***LIBS Methodology***

The laser was pulsed at 1064 nm (CFR200, Quantel) with 8 ns of emission and pulses of 200 mJ. The focus was on 10 μm spot to shoot specimens. The optical emission from the ablated tissue was collected by a six-channel fiber bundle (2000 μm diameter), positioned 35mm from the focal point at 45° from the laser beam. The fibers relayed light to six spectrometers spanning 200 – 900 nm with 0.1 nm resolutions (MX2500+, Ocean Optics). The spectrometer was triggered to acquire 0.9 μs after laser firing and with 1 ms acquisition.

***Statistical analysis by Laser-induced breakdown spectroscopy: Two-way-ANOVA***

The statistical analysis of elements identified by Laser-induced breakdown spectroscopy (LIBS) compared two factors: 1) temperature (cold or hot with and without tea) based on 2) intensity of wavelengths (elemental intensity difference). The groups were named by treatment effect: in vivo tea, in vivo water, in vitro hot-tea, in vitro cold-tea, in vitro hot-water, in vitro cold-water. A two-way ANOVA was used for each analysis. The hypothesis for in vivo experiments was tea treatment in vivo had no effect compared with the control treatment (water consumption). The hypothesis for in vitro experiments was in vitro tea treatments (hot-tea or cold-tea) had no effect compared with the control treatment (hot-water or cold-water).

**RESULTS**


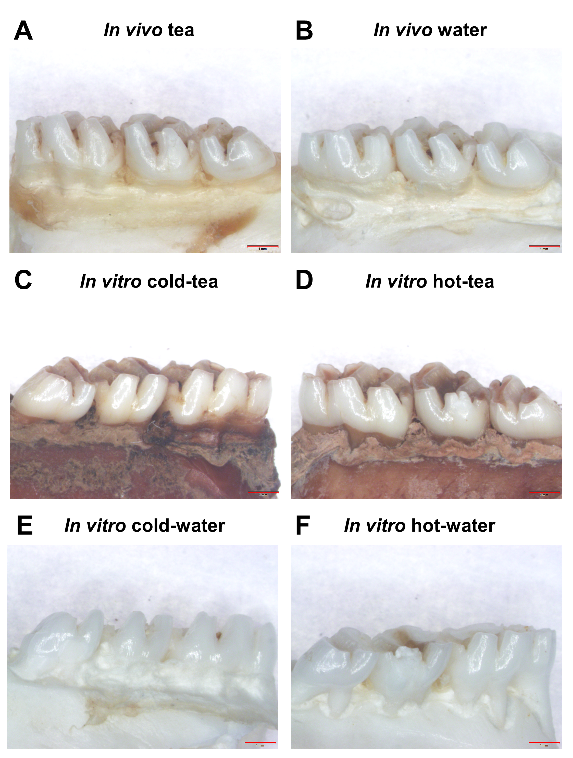


**Figure S-1. Color changes after tea induction.** (A) Teeth from the *in vivo* tea group were slightly more yellow than the control group (B). (C) Teeth from *in vitro* cold-tea did not show a difference in color change compared with the *in vitro* hot-tea group (D). Both *in vitro* cold-tea and *in vitro* hot-tea were dramatically colored brown by tea. Teeth colored by tea were drastically more yellow than cold-water (E) and hot-water (F) groups. Scale bar represents 1 mm (N = 3 jaws, N = 24 teeth per group).

**Statistical analysis of elements identified by Laser-breakdown Spectroscopy (LIBS). ANOVA two-way description.**

ANOVA two-way analysis based on comparison Main comparison of null control hypothesis.

- **In vivo tea vs in vivo water**

For Zn II, groups were significantly different based on intensity of wavelength (d=35, 2736, F = 8.764, P < 0.0001), and green tea treatment (d=1, 2736, F = 1570, P < 0.0001).

For C I, groups were significantly different based on intensity of wavelength (d=18, 1444, F = 39.58, P < 0.0001), and green tea treatment (d=1, 1444, F = 17.76, P < 0.0001).

For P I, groups were significantly different based on intensity of wavelength (d=65, 5016, F = 111.4, P < 0.0001), and green tea treatment (d=1, 5016, F = 31.89, P < 0.0001).

For Mn II, groups were significantly different based on intensity of wavelength (d=19, 1520, F = 44.01, P < 0.0001), but non-significantly different in green tea treatment (d=1, 1520, F = 0.9481, P = 0.3303).

For Mg II, groups were significantly different based on intensity of wavelength (d=61, 4712, F = 182.2, P < 0.0001), and green tea treatment (d=1, 4712, F = 102.7, P < 0.0001).

For Fe I, groups were significantly different based on intensity of wavelength (d=61, 4712, F = 83.87, P < 0.0001), and green tea treatment (d=1, 4712, F = 43.68, P < 0.0001).

For Ca II, groups were significantly different based on intensity of wavelength (d=95, 7296, F = 129.4, P < 0.0001), and green tea treatment (d= 1, 7296, F = 25.90, P < 0.0001).

For Ca I, groups were significantly different based on intensity of wavelength (d=103, 7904, F = 186.1, P < 0.0001), and green tea treatment (d=1, 7904, F = 140.5, P < 0.0001).

For Sr II, groups were significantly different based on intensity of wavelength (d=33, 2584, F = 25.79, P < 0.0001), and green tea treatment (d=1, 2584, F = 243.1, P < 0.0001).

For Na II, groups were significantly different based on intensity of wavelength (d=17, 1368, F = 99.16, P < 0.0001), and green tea treatment (d=1, 1368, F = 111.5, P < 0.0001).

For K I, groups were significantly different based on intensity of wavelength (d=42, 3268, F = 323.0, P < 0.0001), but non-significantly different in green tea treatment (d=1, 3268, F = 0.5420, P = 0.4617).

For O I, groups were significantly different based on intensity of wavelength (d=22, 1748, F = 72.79, P < 0.0001), but non-significantly different in green tea treatment (d=1, 1748, F = 0.5599, P = 0.4544).

**In vitro**

- **Cold-tea vs hot-tea**

For Zn II, intensity of wavelengths was non significantly different (d=35, 2448, F = 0.1874, P > 0.9999), however, tea temperature was significantly different (F (1, 2448) = 59.75, P < 0.0001).

For C I, groups were significantly different based on intensity of wavelength (d=18, 1292, F = 55.66, P < 0.0001), but non-significantly different in tea temperature (d=1, 1292, F = 1.696, P = 0.1930).

For P I, groups were significantly different based on intensity of wavelength (d=65, 4488, F = 57.98, P < 0.0001), but non-significantly different in tea temperature (d=1, 4488, F = 2.054, P = 0.1519).

For Mn II, groups were significantly different based on intensity of wavelength (d=19, 1360, F = 19.02, P < 0.0001), and tea temperature (d=1, 1360, F = 6.713, P = 0.0097).

For Mg II, groups were significantly different based on intensity of wavelength (d=61, 4216, F = 78.52, P < 0.0001), and tea temperature (d=1, 4216, F = 19.61, P < 0.0001).

For Fe I, groups were significantly different based on intensity of wavelength (d=61, 4216, F = 79.20, P < 0.0001), and tea temperature (d=1, 4216) F = 17.50, P < 0.0001).

For Ca II, groups were significantly different based on intensity of wavelength (d=95, 6528, F = 155.0, P < 0.0001), and tea temperature (F (1, 6528) = 18.38, P < 0.0001).

For Ca I, groups were significantly different based on intensity of wavelength (d=103, 7072, F = 97.99, P < 0.0001), and tea temperature (d=1, 7072, F = 47.85, P < 0.0001).

For Sr II, groups were significantly different based on intensity of wavelength (d=33, 2312, F = 15.99, P < 0.0001), and tea temperature (d=1, 2312, F = 30.55, P < 0.0001).

For Na II, groups were significantly different based on intensity of wavelength (d=17, 1224, F = 56.42, P < 0.0001), and tea temperature (d=1, 1224, F = 21.80, P < 0.0001).

For K I, groups were not significantly different based on intensity of wavelength (d=42, 2924, F = 244.9, P < 0.0001), but non-significantly different in tea temperature (1, 2924 F = 3.594, P = 0.0581).

For O I, groups were significantly different based on intensity of wavelength (d=22, 1564, F = 86.00, P < 0.0001), and tea temperature (d=1, 1564, F = 131.3, P < 0.0001).

- **Cold-tea vs cold-water**

For Zn II, groups were significantly different based on intensity of wavelength (d=35, 1692, F = 0.04289, P > 0.9999), but non-significantly different on tea treatment (d=1, 1692, F = 5.357, P = 0.0208).

For C I, groups were significantly different based on intensity of wavelength (d=18, 893, F = 22.81, P < 0.0001), but non-significantly different on tea treatment (d=1, 893, F = 1.265, P = 0.2610).

For P I, groups were significantly different based on intensity of wavelength (d=65, 5478, F = 48.54, P < 0.0001), but non-significantly different on tea treatment (d=2, 5478, F = 1.672, P = 0.1880).

For Mn II, groups were significantly different based on intensity of wavelength (d=19, 940, F = 7.925, P < 0.0001), but non-significantly different on tea treatment (d=1, 940, F = 0.9240, P = 0.3367).

For Mg II, groups were significantly different based on intensity of wavelength (d=61, 2914, F = 21.17, P < 0.0001), and tea treatment (d=1, 2914, F = 89.71, P < 0.0001).

For Fe I, groups were significantly different based on intensity of wavelength (d=61, 2914, F = 14.04, P < 0.0001), and tea treatment (d=1, 2914, F = 19.96, P < 0.0001).

For Ca II, groups were significantly different based on intensity of wavelength (d=95, 4512, F = 44.95, P < 0.0001), and tea treatment (d=1, 4512, F = 505.6, P < 0.0001).

For Ca I, groups were significantly different based on intensity of wavelength (d=103, 4888, F = 44.75, P < 0.0001), and tea treatment (d=1, 4888, F = 163.4, P < 0.0001).

For Sr II, groups were significantly different based on intensity of wavelength (d=33, 1598, F = 4.790, P < 0.0001) but non-significantly different on tea treatment (d=1, 1598, F = 0.9363, P = 0.3334).

For Na II, groups were significantly different based on intensity of wavelength (d=17, 846, F = 27.51, P < 0.0001), but non-significantly different in tea treatment (d=1, 846, F = 6.620, P = 0.0103).

For K I, groups were significantly different based on intensity of wavelength (d=42, 2021, F = 118.0, P < 0.0001), but non-significantly different on tea treatment (d=1, 2021, F = 1.444, P = 0.2296 1).

For O I, groups were significantly different based on intensity of wavelength (d=22, 1081, F = 25.21, P < 0.0001), and tea treatment (d=1, 1081, F = 333.8, P < 0.0001).

- **Hot-tea vs hot-water**

For Zn II, groups were significantly different based on intensity of wavelength (d=35, 1836, F = 4.340, P < 0.0001), and on tea treatment (d=1, 1836, F = 83.24, P < 0.0001).

For C I, groups were significantly different based on intensity of wavelength (d=18, 969, F = 41.97, P < 0.0001), and on tea treatment (d=1, 969, F = 106.2, P < 0.0001).

For P I, groups were significantly different based on intensity of wavelength (d=65, 3366, F = 35.96, P < 0.0001), and on tea treatment (F (1, 3366) = 55.91, P < 0.0001).

For Mn II, groups were significantly different based on intensity of wavelength (d=19, 1020, F = 16.01, P < 0.0001), and on tea treatment (F (1, 1020) = 66.08, P < 0.0001).

For Mg II, groups were significantly different based on intensity of wavelength (d=61, 3162, F = 82.49, P < 0.0001), and on tea treatment (d=1, 3162, F = 251.0, P < 0.0001).

For Fe I, groups were significantly different based on intensity of wavelength (d=61, 3162, F = 54.86, P < 0.0001), and on tea treatment (d=1, 3162) F = 557.7, P < 0.0001).

For Ca II, groups were significantly different based on intensity of wavelength (d=95, 4896, F = 71.18, P < 0.0001), and on tea treatment (d=1, 4896) F = 650.0, P < 0.0001).

For Ca I, groups were significantly different based on intensity of wavelength (d=103, 5304, F = 54.49, P < 0.0001), but non-significantly different on tea treatment (d=1, 5304, F = 0.1841, P = 0.6679).

For Sr II, groups were significantly different based on intensity of wavelength (d=33, 1734, F = 23.60, P < 0.0001), but non-significantly different on tea treatment (d=1, 1734, F = 0.01088, P = 0.9169).

For Na II, groups were significantly different based on intensity of wavelength (d=17, 918, F = 37.14, P < 0.0001), and on tea treatment (F (1, 918) = 31.33, P < 0.0001).

For K I, groups were significantly different based on intensity of wavelength (d=42, 2193, F = 133.2, P < 0.0001), and on tea treatment (d=1, 2193, F = 145.8, P < 0.0001).

For O I, groups were significantly different based on intensity of wavelength (d=22, 1173, F = 32.11, P < 0.0001), and on tea treatment (d=1, 1173, F = 760.9, P < 0.0001).

- **Cold-water vs hot-water**

For Zn II, intensity of wavelength was non significantly different (d=35, 1080, F = 1.363, P = 0.0789), however, temperature was significantly different (d=1, 1080, F = 469.1, P < 0.0001).

For C I, groups were significantly different based on intensity of wavelength (d=18, 570) F = 25.70, P < 0.0001), and temperature (d=1, 570) F = 40.53, P < 0.0001).

For P I, groups were significantly different based on intensity of wavelength (d=65, 1980, F = 43.70, P < 0.0001), and temperature (d=1, 1980, F = 80.91, P < 0.0001).

For Mn II, groups were significantly different based on intensity of wavelength (d=19, 600, F = 20.71, P < 0.0001), and temperature (d=1, 600, F = 37.77, P < 0.0001).

For Mg II, groups were significantly different based on intensity of wavelength (d=61, 1860, F = 41.10, P < 0.0001), and temperature (d=1, 1860, F = 7.641, P = 0.0058).

For Fe I, groups were significantly different based on intensity of wavelength (d=61, 1860, F = 31.98, P < 0.0001), and temperature (d=1, 1860, F = 497.7, P < 0.0001).

For Ca II, groups were significantly different based on intensity of wavelength (d=95, 2880, F = 34.60, P < 0.0001), but non-significantly different based on temperature (d=1, 2880, F = 0.02950, P = 0.8636).

For Ca I, groups were significantly different based on intensity of wavelength (d=103, 3120, F = 44.66, P < 0.0001), and temperature (d=1, 3120) F = 63.54, P < 0.0001).

For Sr II, groups were significantly different based on intensity of wavelength (d=33, 1020, F = 13.40, P < 0.0001), and temperature (d=1, 1020, F = 86.35, P < 0.0001).

For Na II, groups were significantly different based on intensity of wavelength (d=17, 540, F = 21.94, P < 0.0001), but non-significantly different based on temperature (d=1, 540, F = 0.8591, P = 0.3544).

For K I, groups were significantly different based on intensity of wavelength (d=42, 1290, F = 103.3, P < 0.0001), and temperature (d=1, 1290, F = 179.1, P < 0.0001).

For O I, groups were significantly different based on intensity of wavelength (d=22, 690, F = 14.16, P < 0.0001), and temperature (d=1, 690, F = 21.41, P < 0.0001).
