# Supplementary material for: Correction: Spectroscopic and microscopic examination of teeth exposed to green tea at different temperatures
Source: PLoS One. 2025 Dec 12;20(12):e0338964. doi: 10.1371/journal.pone.0338964 (PMC12700374; doi:10.1371/journal.pone.0338964)
Supplement: S1 File — (PDF) [file pone.0338964.s001.pdf]

PLOS ONE

RESEARCH ARTICLE

Spectroscopic and microscopic examination of teeth exposed to green tea at different temperatures

Sinai H. C. Manno[1,2,3☯], Francis A. M. Manno[1,3☯], Li Tian[1,2], Muhammad S. Khan[3], Irfan Ahmed[3,4], Yuanchao Liu[3], Vincent W. T. Li[1], Shisan Xu[1], Fangjing Xie[1], Tak Fu Hung[5], Victor Ma[6], William Cho[6], Beatriz Aldape[7], Shuk Han Cheng[1,2,5]*, Condon Lau[1]*

1 Department of Biomedical Sciences, City University of Hong Kong, Hong Kong SAR, China, **2** State Key Laboratory of Marine Pollution (SKLMP), City University of Hong Kong, Hong Kong SAR, China, **3** Department of Physics, City University of Hong Kong, Hong Kong SAR, China, **4** Department of Electrical Engineering, Sukkur IBA University, Sukkur, Sindh, Pakistan, **5** Department of Materials Science and Engineering, City University of Hong Kong, Hong Kong, Hong Kong SAR, China, **6** Department of Clinical Oncology, Queen Elizabeth Hospital, Hong Kong, Hong Kong SAR, China, **7** División de Estudios de Posgrado e Investigación, Facultad de Odontología, Portal de la Universidad Nacional Autónoma de México, México, D.F., México

☯ These authors contributed equally to this work.
* condon.lau@cityu.edu.hk (CL); bhcheng@cityu.edu.hk (SHC)

OPEN ACCESS

Citation: Manno SHC, Manno FAM, Tian L, Khan MS, Ahmed I, Liu Y, et al. (2020) Spectroscopic and microscopic examination of teeth exposed to green tea at different temperatures. PLoS ONE 15(12): e0244542. https://doi.org/10.1371/journal.pone.0244542

Editor: Richard Johannes Wierichs, Universitat Bern, SWITZERLAND

Received: February 13, 2020

Accepted: December 14, 2020

Published: December 30, 2020

Copyright: © 2020 Manno et al. This is an open access article distributed under the terms of the Creative Commons Attribution License, which permits unrestricted use, distribution, and reproduction in any medium, provided the original author and source are credited.

Data Availability Statement: All relevant data are within the paper and its Supporting Information and Appendix files.

Funding: This work was supported in part by the Hong Kong Research Grants Council (ECS CityU21201217 to CL) and the City University of Hong Kong (9610338, 9610378, and 7005507 to CL). It was also supported by the University of Hong Kong (9610348 to SHC) and CONACYT, CVU 622691 (support number: 739501 to SHCM). The funders had no role in study design, data collection

Abstract

Tea is a popular beverage consumed at different temperatures. The effect of tea on teeth at different temperatures has not been studied previously. The present study used an *in vitro* green tea immersed tooth model at different tea temperatures (hot and cold) compared to an *in vivo* tea administration model allowing rats to drink tea over the course of a week. The elements present in tea leaves were identified by Inductively Coupled Plasma Mass Spectrometry (ICP-MS) and compared to the elements in teeth (enamel surface) using Laser-Induced Breakdown Spectroscopy (LIBS). Here, LIBS demonstrated in vivo and in vitro green tea treatments resulted in a significant increase in the mineral elements found in enamel. For the in vitro assessment, elements in enamel varied based on cold-tea and hot-tea treatment; however, hot water reduced the elements in enamel. Atomic force microscopy found the in vivo tea group had a higher roughness average (RA) compared with the in vivo water group. Cold tea and hot tea in vitro groups demonstrated lower RA than in vitro water controls. Scanning electron microscopy found hot water induced cracks more than 1.3μm in enamel while cold tea and hot tea promoted the adhering of extrinsic matter to teeth. Overall, teeth treated to high temperature lost the mineral phase leading to demineralization. Our results indicate that green tea protects enamel, but its protective action in dental structures is enhanced at cold temperature.

Introduction

Tea is one of the most popular drinks consumed worldwide, especially in China [1, 2]. It has been widely reported that green tea extracts protect dental tissues [3–6]. For example, green-

and analysis, decision to publish, or preparation of the manuscript.

Competing interests: The authors have declared that no competing interests exist.

tea protects against dentin erosion and abrasion [3, 5, 7], periodontal diseases[8], and caries [9]. The protective action of green tea is due to the inhibition of metalloproteinases (MMPs) which degrade matrix in dentin [3]. In fact, green tea extracts have been added to dentifrices due to the high presence of catechins which act as suppressors of oxidative stress in periodontal diseases [8]. Despite the advantages of drinking tea, there is a lack of information regarding the influence/interaction of green tea temperature on dental surfaces. The overall goal of this study was to determine the dental changes elicited by tea using an in vivo and in vitro model. The present study combined biophysical techniques with sensitive spectroscopic analyses and microscopic characterization of dental roughness to determine the effect of tea and temperature on dental structures.

Teeth consist of three mineralized tissues: enamel, dentin and cementum. Enamel is considered the hardest tissue in the body, due to approximately 97% of the mineral phase being composed of calcium-phosphorus in the form of hydroxyapatite (HA) crystals [10]. Other elements are also found in minor quantities in enamel and play an important role in the inorganic structure [10, 11]. The principal function of enamel is to protect dentin, which subsequently protects the neurovascular bundle from physical and chemical effects [12]. One common damage facing teeth is erosion, the progressive chemical dissolution of HA by acid or chelation, without bacteriological contributions [13]. Furthermore, erosive lesions can be caused by teeth being exposed to a solution which is unsaturated with respect to the surface of enamel, resulting in the leeching out of minerals [14]. Erosion of teeth can be influenced by many factors including pH and temperature [15].

Additional factors that have an effect on erosion are time of exposure to the erosive agent [15–17] and dietary habits [18–20]. Despite green tea being preferably consumed at higher temperatures, e.g. 65 to 85˚C [21, 22], there is no information relating temperature, and the subsequent effects on dental structures. For example, the degree of erosion on teeth was proportional to the increase in temperature; however, the assessment was using citric acid [23], a corrosive liquid. In soft drinks, a low pH is a dissolution factor causing strong erosion at a range of different temperatures [15]. Tea in general is acidic; therefore it is interesting to note that black tea possesses a low acid anion profiles and thus, does not influence dental erosion. [24]. Green tea may share similar properties; although, studies have not elucidated its chemical reaction to different pHs and temperatures. A recent study [25], demonstrated in situ that black tea and green tea have a positive effect on enamel and dentin structures, but their assessment did not include temperature parameters. It is known that there exists a correlation between temperature and rate of dissolution of HA eliciting erosion in teeth [15, 23]. To date, there is no information testing whether green tea can protect teeth under different temperature conditions. Unfortunately, when erosion occurs, the progress is difficult to identify accurately in humans since demineralization occurs imperceptibly over time. Therefore, studies using sensitive spectroscopic techniques could aid in deciphering the effect of tea temperature on teeth.

Recently, we demonstrated that spectroscopic techniques have the potential to discriminate elemental changes in teeth treated with coffee [26]. In the present report, we identified the subtle effect that temperature has on teeth treated with tea. To determine the effect of tea temperature on teeth, we quantified elements present in green tea using Inductively Coupled Plasma Mass Spectrometry (ICP-MS) and compared this to Laser-Induced Breakdown Spectroscopy (LIBS) to assess the enamel surface. The LIBS technique has been a useful tool to discriminate and differentiate elements accurately with high spatial resolution [27]. Additionally, LIBS is used for the identification of elements in soft tissues [28–31] and mineralized tissues without invasive sampling preparation [32–36]. In the present study, we have quantified the variation of inorganic elements in teeth subjected to hot tea and cold tea (room temperature—RT) in an

in vitro model using LIBS. Additionally, we combined spectroscopy with microscopy analyses to provide a wide panorama of assessing the effect of tea temperature on teeth. For example, Atomic Force Microscopy (AFM) is a useful instrument that previously had been used to discriminate dental surface damage from soft drinks [15, 37, 38]. To distinguish surface topographical characteristics, AFM is combined with Scanning Electron Microscopy (SEM) which offers high resolution imaging, crucial to identifying dental erosion [15, 39–43]. The principal aim of this study was to investigate the influence of tea temperature on teeth. The following investigation has two questions: 1) Can the elements contained in tea interact with the elements in teeth? 2) How does this interaction vary with temperature? The results provide important information for promoting oral health in preventing erosion and/or demineralization of teeth.

Material and methods

Animal model

The present study included in vivo and in vitro experiments using a total of eighteen Sprague-Dawley (SD) rats (350–450 g). The rats were divided into four in vitro groups and two in vivo groups (6 groups in total: 12 bilateral jaws for in vitro; 6 bilateral jaws for in vivo). Each group contained N = 3 jaws. Each bilateral jaw has N = 8 teeth; therefore, N = 24 teeth per group. A total of 144 teeth were used for both in vitro and in vivo experiments (144 teeth divided by 18 inferior jaws). The animal research ethics committee of the City University of Hong Kong approved all procedures, which were in accordance with the relevant guidelines and regulations of the Department of Health of the Hong Kong Special Administrative Region. Rats were euthanized with CO2 overdose and jaws were extracted. The hard tissues were separated from the adjacent soft tissue and cleaned with fresh water. Incisors and molars were included in this study. All tissues were preserved in sterile water and stored at 4˚C for 24 hrs. Subsequently, samples were washed in distilled water and dried in a freeze dryer (LABCONCO, Catalog No: 7806031) for 48 hours. Fig 1 illustrates the experimental design for tea preparation, in vivo and in vitro models, and the instrumentation used.

Back-of-the-envelope calculations

We simulated drinking tea for a 5-year period daily for five minutes using a week immersion paradigm based on a back-of-the-envelope estimation inspired by E. Fermi [26]. For in vitro experiments, teeth were immersed for one week in a hot or room temperature (cold) solution of green tea or water. For the in vivo assessment, rats were provided with one week of green tea solution or water ad libitum. The shorter time frame for the in vivo experiments was due to rodents' hyperactivity (i.e. caffeine from tea).

Tea preparation

Green teabags were used from a commercial brand in China (Luk Yu Tea). The concentration was 10 g of green tea (equivalent to 5 teabags) per 250 ml of water. The preparation of tea was repeated every 24 hours for both in vivo and in vitro experiments. The pH of tea was evaluated using the Eutech-Instrument-700 pH meter. For the hot-tea group, 50 ml of tea solution was prepared and maintained at 65˚C in a mini cooler (Major Science, MC-0203), with the pH subsequently calculated. The pH was calculated as the average of ten room temperature (25˚C) and hot tea examinations. The pH of water at 25˚C or 65˚C was also measured. A detailed explanation of tea preparation is found in the Supporting Information.

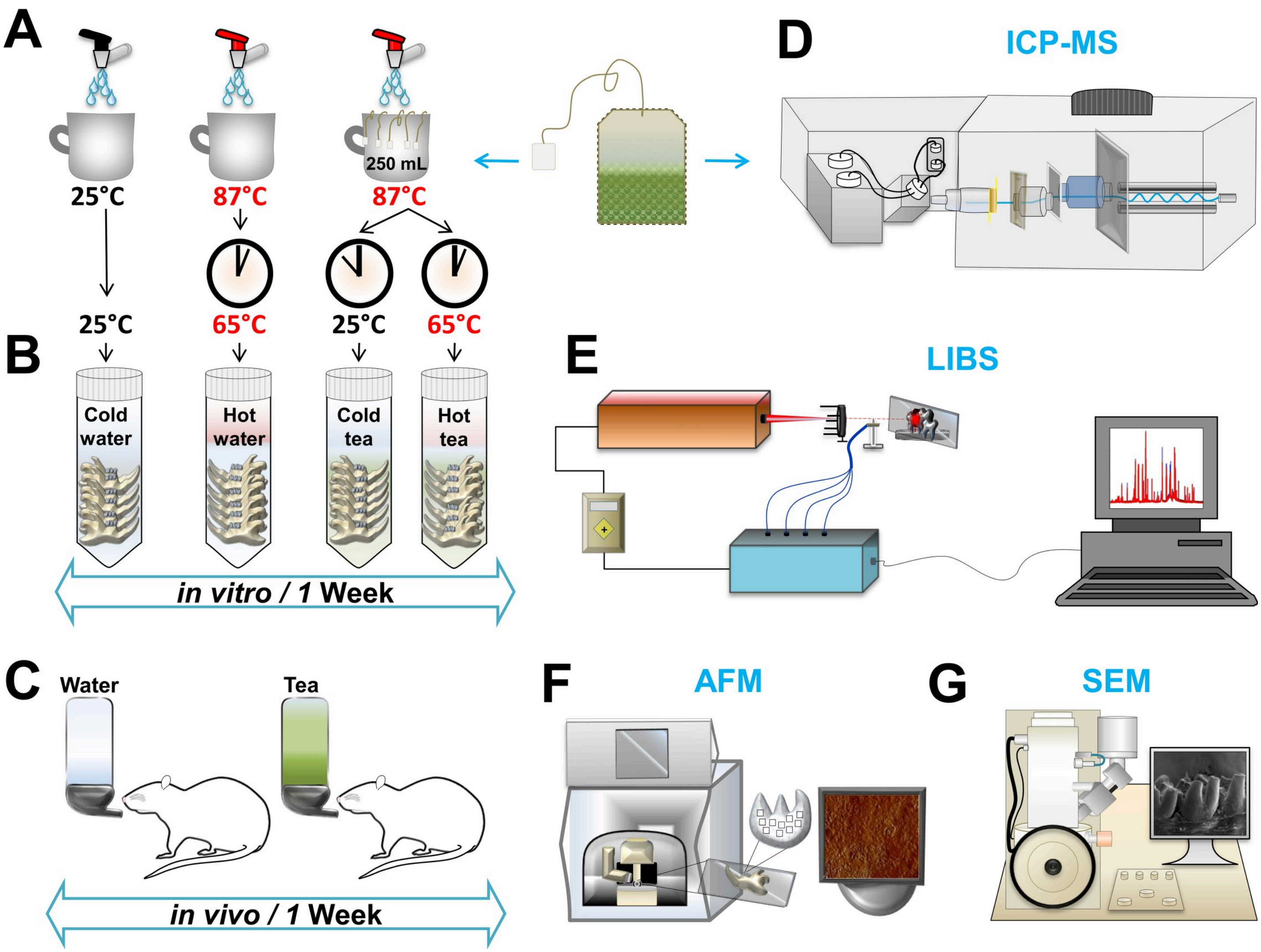

Fig 1. Experimental design. (**A**) The preparation of tea consisted of dissolving five teabags in 250 mL hot-water at 87˚C. For the hot-tea group, teeth were immersed after tea preparation. For the cold-tea group, tea was allowed to cool to room temperature and then teeth were immersed. For control groups, water was made hot or allowed to cool to room temperature as in the tea groups. (**B**) Jaws were immersed into cold-water, hot-water, cold-tea, hot-tea. The procedure was repeated daily for one week. (**C**) The preparation of tea or water was provided to rats for in vivo consumption. (**D**) The analysis of elemental content in green tea was evaluated by Inductively Coupled Plasma Mass Spectrometry (ICP-MS). (**E**) The elemental composition of enamel was evaluated by using Laser-Induced Breakdown Spectroscopy (LIBS). (**F**) Atomic Force Microscopy (AFM) was used to identify roughness on the enamel surface. (**G**) Scanning Electron Microscopy (SEM) was used to characterize dental surface ultrastructure.

https://doi.org/10.1371/journal.pone.0244542.g001

Inductively Coupled Plasma Mass Spectrometry (ICP-MS)

The principal metals present in green tea were determined using the AOAC 999.10 protocol [44] by Inductive Coupled Plasma Mass Spectrometer (ICP-MS-Agilent 7500cs). We quantified the concentration of Ca, K, Mg, Mn, Zn, Fe, and Na in 50 g of tea powder.

Laser-Induced Breakdown Spectroscopy (LIBS)

The LIBS procedure was similar to our previous method examining the effect of coffee on teeth [26]. The experiments were performed in standard atmospheric air. Dental tissues from three regions of the crown were utilized (incisal, occlusal and medial lobules) and three laser

pulses were fired per specimen. The emission spectra were processed to generate a graph showing the intensity per wavelength. Every emission was computer controlled to expedite data acquisition. Each acquired spectrum was independently baseline subtracted. The representative graphs were analyzed and processed in Origin Pro 8.5. Ratios were measured per sample in proportion to calcium lines and analyzed based on previous reports [45–49]; P/Ca; Mg/Ca; Zn/Ca; Sr/Ca and C/Ca. More detailed analysis for LIBS is described in the S1 File.

Atomic Force Microscopy (AFM)

Jaws containing molars were placed in a glass holder to examine topographical surfaces using an Atomic Force Microscope (Dimention-ICON-Scan Asyst NanoScope®V). Four regions were randomly selected per tooth from the crown surface (incisal, occlusal and medial lobules). A scanning rate of 1Hz was utilized covering an area of 10μm^2. The piezoelectric scanner resolution was 256x256 pixels with a Z-range limit of 12.5μm. The regions were randomly selected from the enamel rods. The data was analyzed using NanoScope Analysis v1.40R1 to determine the roughness average (RA). Images generated were used to determine the peak force error, the topographic surface, the processed 3-D image and the histogram depth.

Scanning Electron Microscopy (SEM)

The jaws were separated from the incisor and placed in a holder with carbon tape, and placed under vacuum for 72 hours. Subsequently, the specimens were coated with gold. We used scanning electron microscopy (SEM; JEOL JSM-820) with an acceleration voltage of 20 kV beam irradiation of secondary electrons and a pressure of 1x10^{-5} Pa. The magnification proceeded from 20x, 100x, 500x, 1000x, 2000x to 8000x. Images were captured from four regions: 1) occlusal zone from molars, 2) buccal groove zone from molars, 3) mesial or distal areas from molars, and 4) incisal ridge from inferior incisors.

Statistics

Tea groups were compared to their respective control water treatments. The groups were named by treatment effect: in vivo tea, in vivo water, in vitro hot-tea, in vitro cold-tea, in vitro hot-water, and, in vitro cold-water. The statistical analysis was based on a two-way ANOVA, with follow-up t-test using the Holm-Sidak method, with a critical value for $p < 0.05$. An unpaired t-test analysis with Welch's correction was also used when appropriate to compare groups. All statistical analysis was performed using GraphPad Prism 6.0. The primary null hypothesis was tea treatment in vivo had no effect compared with the control treatment. The secondary null hypothesis was in vitro treatments of hot or cold-water had no effect compared to tea treatments of hot tea or cold-tea. For more details, see Supporting Information.

Results

Fig 1, shows the study design, illustrating how tea was prepared for the in vitro and in vivo assessments. Changes in color were more substantial in the in vitro assessment than in vivo observations (S1 Fig in S1 File).

The water had a pH 7.92 at cold temperature (20˚C) and pH 7.71 at hot temperature (65˚C). The pH of tea had a value of 5.45 in cold conditions, while at 65˚C the pH dropped to 5.32. The analysis of elemental content present in green tea leaves assessed by ICP-MS revealed high concentrations of Ca, Mg, K, and Mn; while Zn, Fe, and Na were present in minor amounts (Table 1). For in vivo experiments, the consumption of tea or water did not vary between groups. Rats ingested ≈20–50 ml a day (10 ml/100 g body weight/day).

Table 1. Elemental content in tea leaves identified by ICP-MS.

Tea sample (Element)	Content (mg/kg)	Relative Standard Deviation (RSD%)
**K**	14806.60 mg/kg	0.5
**Ca**	3735.14 mg/kg	2.3
**Mg**	2243.35 mg/kg	1.2
**Mn**	1149.58 mg/kg	0.5
**Fe**	196.44 mg/kg	0.6
**Zn**	33.09 mg/kg	1.1
**Na**	10.31 mg/kg	0.1

Values expressed in mg/kg from 50 mg green tea leaves analysis. Values are expressed with its Relative Standard Deviation (RSD%).

https://doi.org/10.1371/journal.pone.0244542.t001

LIBS elemental analysis

To identify whether drinking tea at different temperatures could alter enamel, we analyzed the elemental concentration by LIBS. The analysis was based on the standard NIST atomic emission database for the identification of Zn, C, Ca, P, Mg, Mn, Na, Sr, Fe, K and O. The intensity of those elements between groups is shown in Fig 2. Comparison of ratios from specific elements is shown in Table 2.

In vivo. The in vivo tea group showed a generalized increase in the main elements forming the mineral phase of enamel (Fig 2A and 2B). The two-way ANOVA demonstrated that green tea interacted with specific elements forming enamel in the in vivo model (see Supporting Information). The T-test with Holm-Sidak method, demonstrated that intensities of Ca, P, Mg, Mn, Sr, Fe and K lines were statistically increased for the in vivo tea group compared with the in vivo water control group (Fig 2A, S1 Appendix). A heatmap represents the visualization of intensity differences for the in vivo groups (Fig 2B; S2 and S3 Appendices). The ratios of elements P/Ca, Zn/Ca ($P<0.01$, or $P<0.0001$), Mg/Ca ($P<0.0001$), and C/Ca ($P<0.05$) were statistically significantly different between in vivo tea and in vivo water (Table 2).

In vitro. Fig 2C–2G represents the in vitro treatment of hot or cold green tea using LIBS to assess elemental characteristics of enamel. The analysis from the in vitro groups were performed between tea or water, cold or hot. A two-way ANOVA demonstrated that in vitro green tea interacted with the elements in enamel (see S2 File). The Holm-Sidak method was performed to compare groups to identify specific elements in enamel which might have changed due to tea or temperature (S1 Appendix) and the ratio of elements of interest (Table 2).

There was no statistically significant difference in the intensity between cold-tea and hot-tea treatments between elements (Fig 2C). The hot-tea group had a higher intensity in P, Mn, Fe, K and O; while Ca, Mg, Sr and Na increased from cold-tea, although none of them reached significance (S1 File, S1 Appendix). A small significance ($P<0.05$) in the Zn/Ca ratio was noticed in the cold-tea comparison with hot-tea (Table 2). A general increase in the elements in enamel was seen in the cold-tea group compared to the cold-water group (Fig 2D). A statistically significant increase in Ca and O in cold-tea was found compared with cold-water (Fig 2D; S1 Appendix). The ratio of elements for cold-tea and cold-water was significantly different for P/Ca, Mg/Ca ($P<0.0001$), and C/Ca ($P<0.01$), (Table 2). The hot-tea treatment revealed a generalized increase in elemental intensities compared to hot-water (Fig 2E). A significant increase in C, Mg, Ca, P, Fe, K and O in the hot-tea group was found compared to hot-water (S1 Appendix). The ratio of elements for hot-tea and hot-water was significantly different for Mg/Ca and Sr/Ca ($P<0.0001$), while a small difference existed for P/Ca and Zn/Ca ($P<0.05$;

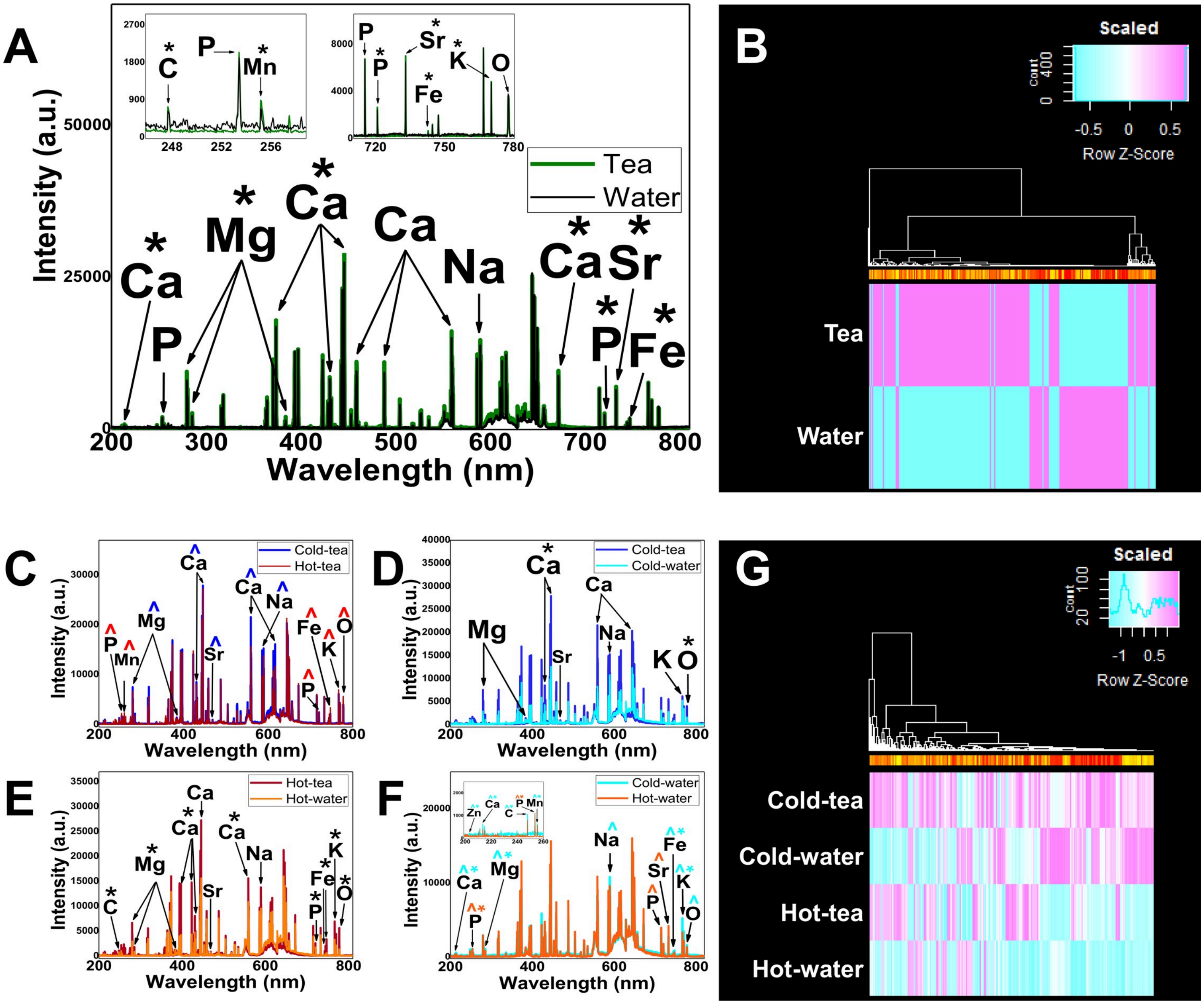

Fig 2. LIBS demonstrates changes in enamel composition. (**A**) The comparison of emission lines from *in vivo* teeth treated with tea (green lines) and water (black lines). The insert shows the range of wavelengths (200–260 nm, and 700–780 nm) displaying the specific element peaks from *in vivo* groups. (**B**) Heat map visualization comparing *in vivo* tea vs *in vivo* water. (**C**) Comparison between in vitro groups, Cold-tea (blue lines) vs hot-tea (red lines) showing the elemental intensity variation. (**D**) Cold-tea (dark blue lines) vs cold-water (light blue lines) comparison showing a significant increase in Ca and O lines from cold-tea group. (**E**) Hot-tea (red lines) vs hot-water (orange lines) showing a significant increase in the elements from hot-tea. (**F**) Hot-water (orange lines) vs cold-water (light blue lines) showing a significant increase in the elements from both groups. Insert shows the range of wavelengths (200–260 nm) displaying the specific element peaks from control hot and cold water. Statistically significant increases are represented by *. The major intensities are represented by ^'s colored coded the same as the group color line. (**G**) Heat map visualization for in vitro groups. For both heatmaps the color is a representation of the difference in intensity between groups. Heat map is a representation of difference where 0 is zero difference, scaled from -1 to 1 representing the range of differences. The color is a representation of change difference. The clustering is based on likeness for the different peaks from the wavelengths.

https://doi.org/10.1371/journal.pone.0244542.g002

Table 2). The cold-water group had a significant increase in Zn, Ca, C, Mn, Mg, Fe and K compared with the hot-water group (Fig 2F; S1 Appendix). The intensity of Na was increased in cold-water compared to the hot-water group, although it did not reach statistical significance. Hot-water revealed a statistically significant increase in P intensity compared to the cold-water

Table 2. Representative elemental ratios for enamel based on emission peaks using LIBS.

In vivo Tea vs *In vivo* water						
**Analyte line (nm)/Reference line (nm)**	**Tea Ratio (mean±SD)**	**Water Ratio (mean±SD)**	**Significance**	**P value**	**t ratio**	**df =**
P (215.27)/Ca (211.29)	0.226±0.267	0.548±0.429	**	0.00314962	3.1028	50
Mg (383.02)/Ca (393.45)	0.045±0.035	0.013±0.007	***	< 0.0001	4.8927	50
Zn (202.52)/Ca (317.92)	0.038±0.037	0.094±0.055	**	0.000136092	4.133	50
Zn (206.45)/Ca (211.29)	0.284±0.392	1.109±0.733	***	< 0.0001	4.7921	50
Sr (460.75)/Ca (458.59)	0.377±0.117	0.315±0.125	NS	0.0755372	1.8149	50
C (247.71)/Ca (317.92)	0.035±0.030	0.060±0.046	*	0.0307674	2.2229	50
**Cold-tea vs Hot-tea**						
**Analyte line (nm)/ Reference line (nm)**	**Cold-tea Ratio (mean±SD)**	**Hot-tea Ratio (mean±SD)**	**Significance**	**P value**	**t ratio**	**df =**
P (215.27)/Ca (211.29)	0.250±0.227	0.254±0.258	NS	0.956719	0.05459	42
Mg (383.02)/Ca (393.45)	0.037±0.032	0.029±0.010	NS	0.269403	1.1192	42
Zn (202.52)/Ca (317.92)	0.062±0.139	0.049±0.061	NS	0.689947	0.40169	42
Zn (206.45)/Ca (211.29)	0.423±0.437	0.210±0.162	*	0.0378992	2.1436	42
Sr (460.75)/Ca (458.59)	0.493±0.237	0.526±0.215	NS	0.631102	0.48371	42
C (247.71)/Ca (317.92)	0.075±0.145	0.073±0.088	NS	0.956156	0.05530	42
**Cold-tea vs Cold-water**						
**Analyte line (nm)/ Reference line (nm)**	**Cold-tea Ratio (mean±SD)**	**Cold-water Ratio (mean±SD)**	**Significance**	**P value**	**t ratio**	**df =**
P (215.27)/Ca (211.29)	0.250±0.227	0.704±0.318	***	< 0.0001	5.0016	34
Mg (383.02)/Ca (393.45)	0.037±0.032	0.222±0.122	***	< 0.0001	6.8048	34
Zn (202.52)/Ca (317.92)	0.062±0.139	0.130±0.149	NS	0.173017	1.3918	34
Zn (206.45)/Ca (211.29)	0.423±0.437	0.417±0.315	NS	0.964806	0.04444	34
Sr (460.75)/Ca (458.59)	0.493±0.237	0.523±0.283	NS	0.733448	0.34335	34
C (247.71)/Ca (317.92)	0.075±0.145	0.375±0.375	**	0.00175411	3.3962	34
**Hot-tea vs Hot-water**						
**Analyte line (nm)/ Reference line (nm)**	**Hot-tea Ratio (mean±SD)**	**Hot-water Ratio (mean±SD)**	**Significance**	**P value**	**t ratio**	**df**
P (215.27)/Ca (211.29)	0.254±0.258	0.643±0.739	*	0.0292649	2.2759	34
Mg (383.02)/Ca (393.45)	0.029±0.010	0.132±0.034	***	< 0.0001	13.422	34
Zn (202.52)/Ca (317.92)	0.049±0.061	0.194±0.286	*	0.0267997	2.3147	34
Zn (206.45)/Ca (211.29)	0.210±0.162	0.558±0.685	*	0.027623	2.3014	34
Sr (460.75)/Ca (458.59)	0.526±0.215	0.205±0.031	***	< 0.0001	5.5213	34
C (247.71)/Ca (317.92)	0.073±0.088	0.125±0.202	NS	0.294242	1.0653	34
**Cold-water vs Hot-water**						
**Analyte line (nm)/ Reference line (nm)**	**Cold-water Ratio (mean±SD)**	**Hot-water Ratio (mean±SD)**	**Significance**	**P value**	**t ratio**	**df =**
P (215.27)/Ca (211.29)	0.704±0.318	0.643±0.739	NS	0.778887	0.283	26
Mg (383.02)/Ca (393.45)	0.222±0.122	0.132±0.034	*	0.0132384	2.6589	26
Zn (202.52)/Ca (317.92)	0.130±0.149	0.194±0.286	NS	0.464402	0.74256	26
Zn (206.45)/Ca (211.29)	0.417±0.315	0.558±0.685	NS	0.490303	0.69974	26
Sr (460.75)/Ca (458.59)	0.523±0.283	0.205±0.031	**	0.000292421	4.1794	26
C (247.71)/Ca (317.92)	0.375±0.375	0.125±0.202	*	0.0372031	2.1960	26

Values expressed in Mean ± SD. Calculations from the emission detected by LIBS. Statistical analysis based on a T-test. Statistical significance determined using the Holm-Sidak method, with

* for $p < 0.05$

** for $p < 0.01$

*** for $p < 0.0001$; NS = non-significant; df = degree of freedom.

https://doi.org/10.1371/journal.pone.0244542.t002

group (S1 Appendix). The intensity of Sr was increased in hot-water compared to the cold-water group, although it did not reach statistical significance. The ratio of elements for cold-water and hot-water were statistically significant in Sr/Ca ($P<0.001$), Mg/Ca and C/Ca ($P<0.05$) (Table 2). A heatmap represents the visualization of intensity differences for the in vitro groups (Fig 2G; S2 and S4 Appendices).

Surface enamel roughness

We evaluated the topographical surface and roughness average (RA) of enamel using AFM (Fig 3, Table 3). The comparison among groups indicated differences in roughness and aggregates over the enamel for in vitro treatments. Significantly higher RA was found in the in vivo tea group compared with the in vivo water group ($F = 31.75$, $P<0.0001$; Table 3). For the in vitro treatment, an extrinsic matter appeared deposited over the enamel in cold-tea and hot-tea groups (Fig 3C & 3E). The aggregates were not seen in the control cold-water or hot-water groups (Fig 3D & 3F). Cold-tea was not statistically different from cold-water in RA ($F = 2.145$, $P = 0.2709$). Hot-tea demonstrated a statistically significant lower RA compared to hot-water ($F = 283.4$, $P<0.0001$). Cold-water RA was not significantly different than hot-water ($F = 1.365$, $P = 0.365$). Overall, in vivo tea induced significant roughness over its control, compared to in vitro treatments. The roughness average for in vitro groups and their comparisons can be found in Table 3. Interestingly for the in vitro treatment, green tea had less RA than its controls, although some surface aggregates (Fig 3).

Scanning electron microscopy

SEM was performed analyzing molars and mandibular incisors from the in vivo (Fig 4A and 4B) and in vitro groups (Fig 4C–4F). Overall, we did not observe significant effect on molars (Fig 4A, 4Aa1, 4Aa2 and 4B, 4Bb1, 4Bb2) and incisors (Fig 4A, 4Aa3, 4Aa4 and 4B, 4Bb3, 4Bb4) from either of the in vivo groups. From the captioned molar of the in vivo tea group (Fig 4A and 4Aa1), there is loss of continuity in the vestibular edge allowing the observation of exposed prisms in the secondary enamel. This could be due to the animal's chewing action, bruxism resulting from caffeine in tea.

The in vitro groups treated with tea or water at different temperatures induced several changes in enamel. The cold-tea group (Fig 4C) did not show significant damage in enamel exposure. However, similar to AFM results, the SEM showed that molars of cold-tea had generalized deposition of extrinsic matter along the vestibule-occlusal area (Fig 4C and 4Cc1) and outermost enamel (Fig 4C and 4Cc2). From the incisor, the outer enamel presented a smooth surface with less aggregates (Fig 4C and 4Cc3), although it was not noticeable from the incisor edge (Fig 4C and 4Cc4). Likewise, the hot-tea group showed deposition of extrinsic matter throughout molars (Fig 4D, 4Dd1 and 4Dd2). A portion of dentin exposed from incisors demonstrated that aggregates were deposited over dentinal tubules (Fig 4D and 4Dd3) and outer structures (Fig 4D, 4Dd4 and 4Dd5). In general, the hot-tea group surface was irregular and highly studded with an accumulation of residue over the enamel surface compared to cold-tea. We did not observe significant effect on molars (Fig 4E, 4Ee1 and 4Ee2) and incisors (Fig 4E, 4Ee3 and 4Ee4) from the cold-water group. In contrast, the molars in the hot-water group exhibited noticeable fractures and a severe detachment along the exterior enamel (Fig 4F, 4Ff1 and 4Ff2). At high magnification, major cracks were found of 1.33μm and 1.76μm thickness along the molar groove (Fig 4F and 4Ff3). The incisor in the hot-water group had a flat surface with a polished appearance on the enamel edge (Fig 4F and 4Ff4) and the incisal edge (Fig 4F, 4Ff4 and 4Ff5). Lastly, comparing hot-tea with hot-water, aggregates were observed in the tea group, but not the water group (Fig 4D vs. 4F). Similarly, comparing cold-tea with cold-water,

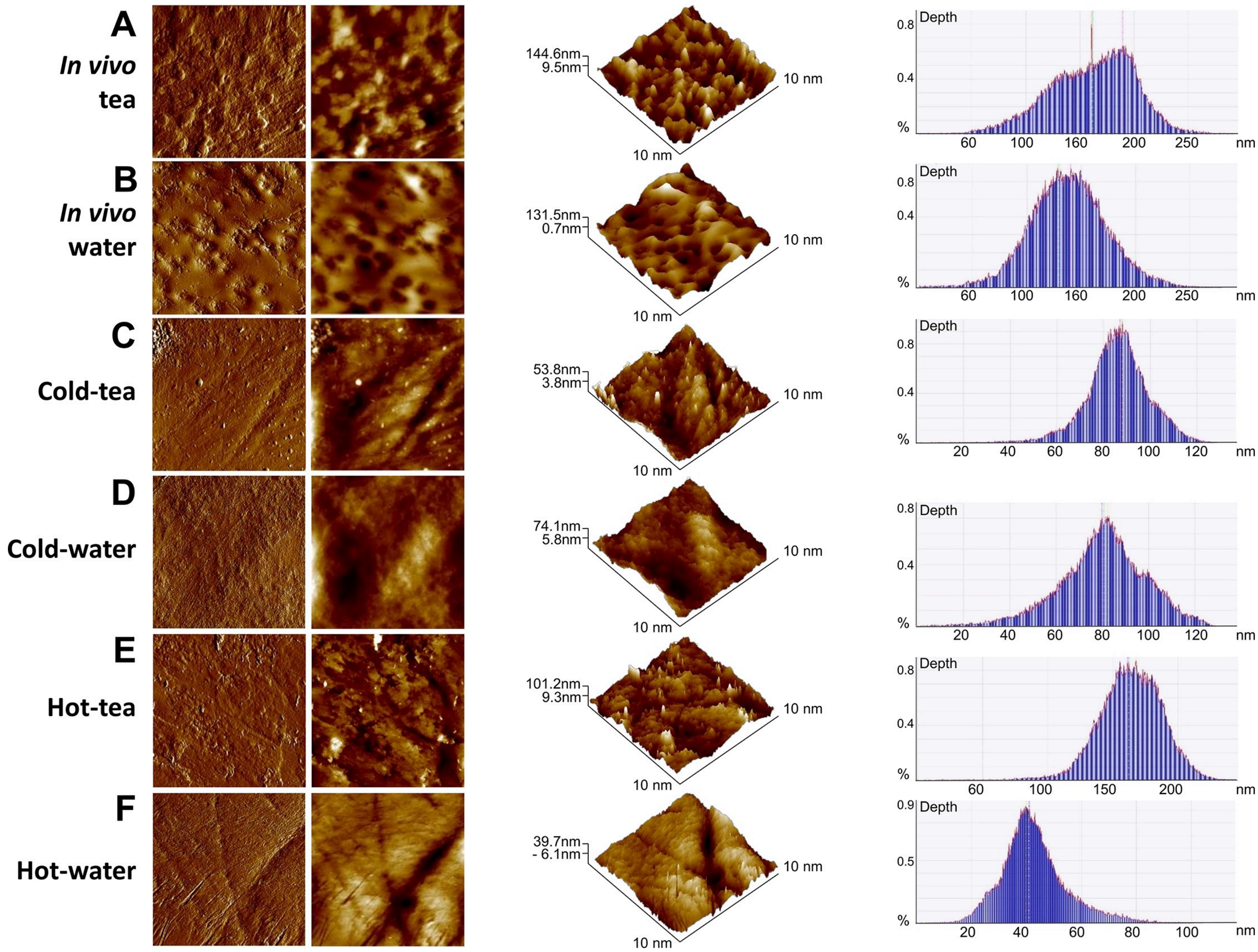

Fig 3. Atomic force microscopy identified loss of roughness on enamel surface induced by high temperature. Representative topographic cleared and flatten images of enamel surface (two columns at the left); three-dimensional images (middle) and depth histograms (right) from in vivo (**A**-**B**), and in vitro groups (**C**-**F**). (**A**) Enamel of teeth from the in vivo tea group showed considerable roughness. (**B**) The in vivo control group treated with water had slightly reduced enamel roughness compared to tea. (**C**) Cold-tea group showed a surface with the appearance of aggregates on the enamel, not seen in the cold-water treatment (**D**). (**E**) Hot-tea showed aggregates over the enamel not seen from hot-water group (**F**). Images were obtained from an 10x10 μm^2 area (N = 3 jaws, N = 24 teeth per group).

https://doi.org/10.1371/journal.pone.0244542.g003

aggregates were observed in the tea group only (Fig 4C vs. 4E). Overall, the SEM analysis demonstrated that higher temperature affected enamel.

Discussion

The current study investigated the effect of green tea on teeth at different temperatures using spectroscopic and microscopic techniques. We originally hypothesized that temperature may reduce the capacity of green tea to protect enamel. Here, we demonstrated that high temperature affects enamel composition likely inducing erosion; but more importantly, green tea can lessen this damage. The protection offered by tea is enhanced at cold temperature. Fig 5 is a schematic model illustrating the effect of tea and temperature on enamel.

Table 3. Enamel roughness after treatment of green tea or water at different temperatures.

Group comparison	RA	Significance	P value	t ratio	df =
In vivo tea	33.65±15.67	***	< 0.0001	1.668	18
In vivo water	25.25± 2.78				
Cold-tea	18.36±7.15	NS	0.2709	1.790	18
Cold-water	25.54±10.47				
Hot-tea	15.65±4.53	***	< 0.0001	1.047	18
Hot-water	18.629±8.96				
Cold-tea	18.36±7.15	NS	0.6507	1.585	18
Hot-water	18.629±8.96				

Roughness Average (RA) (Mean ± SD) measured from 10x10μm^2 area by AFM. Statistical analysis based on unpaired T-test; with Welch's correction

*** for $p < 0.0001$; NS = non-significance; df = degree of freedom.

https://doi.org/10.1371/journal.pone.0244542.t003

Biomechanism of green tea protection

Green tea protects teeth because it contains high amounts of polyphenol catechins. These inhibit the enzymatic reaction of MMPs, the proteins associated with erosion, especially in dentin [3, 5, 9, 50]. High temperature conditions could contribute to the activation of MMPs, exposing the matrix to mineral loss. Using laser induced breakdown spectroscopy (LIBS), we identified that at high temperature, enamel loses the crucial elements that maintain the mineral phase. For example, comparing the hot-water and cold-water groups (Fig 2F), we found a reduction in mineral elements (e.g. Ca) in the hot-water group. Similarly, when we compared the hot-tea versus the hot-water group (Fig 2E), we also found a reduction in the hot-water group. This evidence supports the hypothesis that temperature facilitates the dissolution of hydroxyapatite crystals inducing erosion [15], but tea protects, possibly by preventing dissolution of hydrogen bonds in the hydroxyapatite structure [51].

Tea protects teeth against erosive demineralization [3–7, 9] because the chemical structure of hydroxyapatite (HA), which comprises enamel, is tolerant to substitution by different trace elements [51, 52]. Reports using liquid solutions [53, 54] have shown that Zn, Ca, Mg, Fe, K, and Cu, which are found in tea, are involved in tooth staining. Likewise, bivalent metals such as Sr^{+2} are incorporated into the HA structure when Ca is lost [46, 51, 55–57]. We observed with LIBS that the Sr/Ca ratio from hot-tea was highly increased compared with hot-water. This suggests that Sr ions from tea solution compensate for the loss of Ca because of increased temperature. In general, teeth uptake Sr molecules to compensate HA's structure in response to thermal stress and calcium loss, irrespective of the presence of tea. For example, when comparing cold-water to hot-water groups, Sr/Ca ratio was significantly decreased in hot-water. Humphrey et al., [58], also found that areas having lower Sr/Ca ratio are more mineralized in comparison to areas with higher Sr/Ca.

Microscopic surface features and demineralization

Hard tissues can be topographically characterized by microscopy. There are numerous studies analyzing the effect of acid drinks on enamel and dentin using AFM [15, 37, 38, 59–61]. Augmentation of roughness average (RA) is observed when demineralization is induced by acid drinks [38] or after treatment with bleaching agents [62–64]. However, we do not associate the increased RA from in vivo tea with demineralization because of the well-known protection of teeth by green tea [3–5]. Instead, increased RA may reflect more microorganisms adhering to the tooth surface [65–67]. Tea contains organic residues that facilitate bacterial and plaque

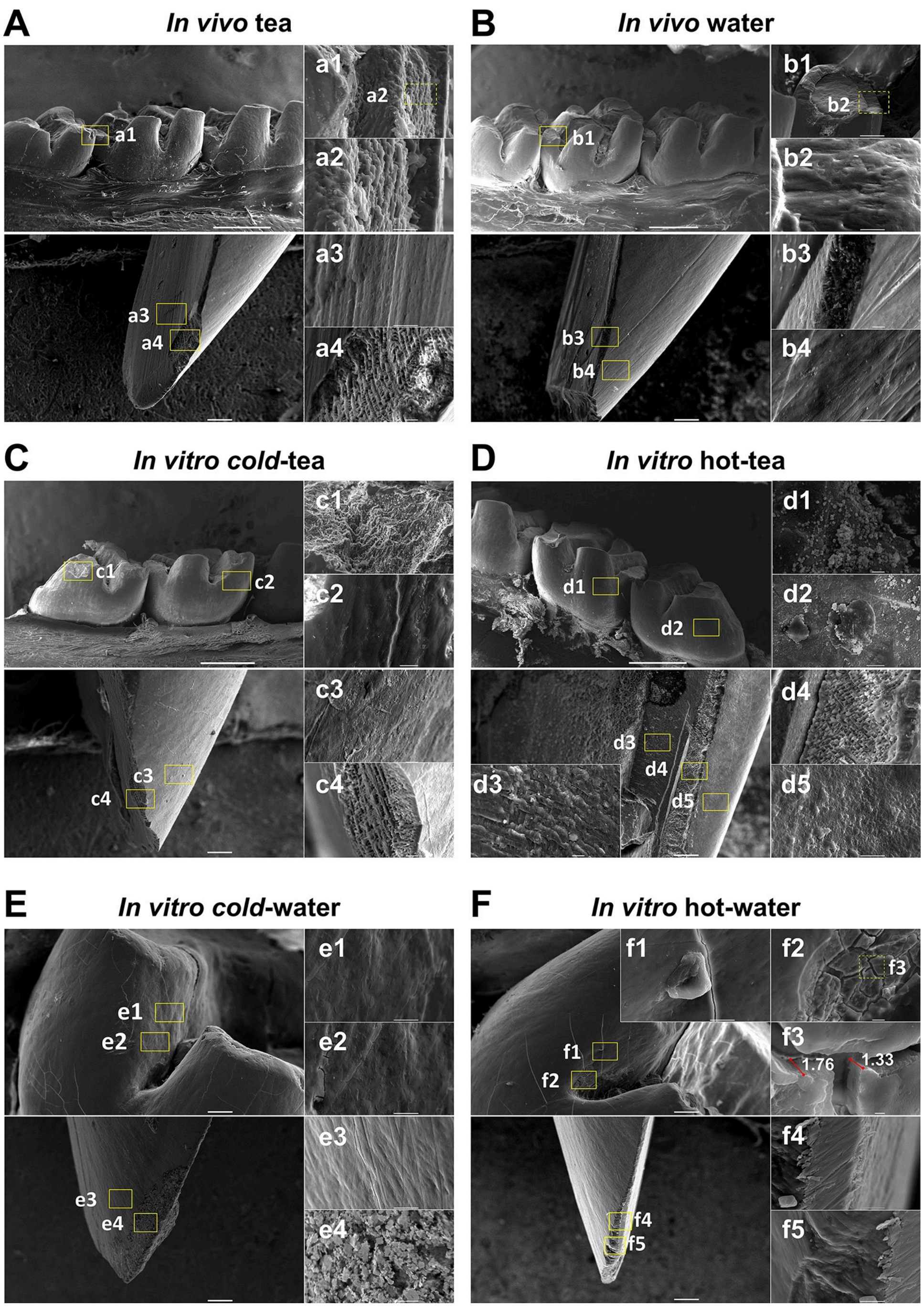

Fig 4. The ultrastructure of enamel is altered by tea and temperature. Molars (upper) and incisors (below) were analyzed by Scanning Electron Microscopy from in vivo **(A-B)** and in vitro groups **(C-F)**. (**A**) For the in vivo tea group, the arrangement of enamel rods from a fracture **(a1, a2)** and the view of the lingual fossa of an incisor showing the normal porosity between hydroxyapatite crystals **(a3)** and dentinal tubules from the apposed surface **(a4)**. (**B**) Molar from the in vivo water group showing the occlusal enamel composition **(b1, b2).** The incisor displays the enamel, dentin junction (**b3**) and the vestibular lobe (**b4**). **(C)** Molar from the cold-tea group showing aggregates over the enamel prisms, and an arrangement, which was not seen in the enamel rods of the incisor from the external lobe **(c3)** and enamel-dentin junction (**c4**). (**D**) In vitro hot-tea group showing a generalized aggregated matter deposited on the surface of the enamel on molars **(d1, d2),** and three regions from an incisor showing roughness of dentinal tubules with deposition of some particles **(d3)**. Some deposition was noticeable in the dentin-enamel junction **(d4).** Deposition was irregularly studded in the outermost enamel of incisor in the hot-tea group **(d5)**. (**E**) The molar from the cold-water group did not show abnormal roughness in the external enamel **(e1, e2)** or in the enamel from the

incisor of the vestibular lobe **(e3)**. Further, some crystal formation from immature enamel was identified **(e4)**. **(F)** The hot-water group induced severe damage in teeth, leading to 1.3 and 1.76 μm fractures and breakages **(f1, f2)** in the upper enamel surface **(f3)** of molars, while some breaks were seen in incisors (**f4, f5**). The scale of Figs is represented by a grey bar 1 mm (main insert), 10 μm (sub-insert) and 1 μm (farthest bottom right panels). Different areas were randomly analyzed per molar or incisor (N = 3 jaws, N = 24 teeth per group).

https://doi.org/10.1371/journal.pone.0244542.g004

formation. In contrast, the in vitro tea groups (cold and hot) showed RA decrease compared to their respective controls. This may be due partly to a reduced microorganism friendly in vitro environment, and protective effects of tea against demineralization. In contrast, the in vitro tea groups had lower RA than the water groups. Hemingway, et al., [68] using optical profilometry, found a similar pattern in teeth treated with different fruit juices at 36˚C, although the degree of abrasion was exacerbated with tooth brushing. This data suggests that aggregates from tea have been deposited in areas where organic matrix is exposed.

We corroborated the features from AFM, with microstructural analysis by SEM. The exposed prisms from the in vivo groups (tea and water) had similar patterns. The in vitro tea and water groups, however, had significant surface differences. Generally, hot-tea and cold-tea had matter adhering along the enamel on molars and incisors. Since hygienic factors were excluded from this study (e.g. brushing), those aggregates were an extrinsic deposition from tea. This is supported by the significantly higher intensity of carbon (from tea leaves) seen by LIBS from in the vivo tea group compared to water, and hot-tea group compared to hot-water. A recent report [25] demonstrated the protective effect of black and green tea and "macromolecular deposits" after black tea treatment only, although the analysis focused on dentin tubules. In this regard, the protective effect of tea as an extrinsic matter likely applies to the enamel.

The outermost layer of enamel in hot-water samples observed by SEM, experienced 1.5 μm cracks which were not found in other groups. Investigation into the demineralization process performed by Lechner, et al., [60] indicated 1μm deep grooves in teeth can be induced by acid in soft drinks. In this study, and similarly by Barbour et al., [15], temperature and exposure time are crucial to modify conditions of teeth. A possible explanation for the cracks focuses on

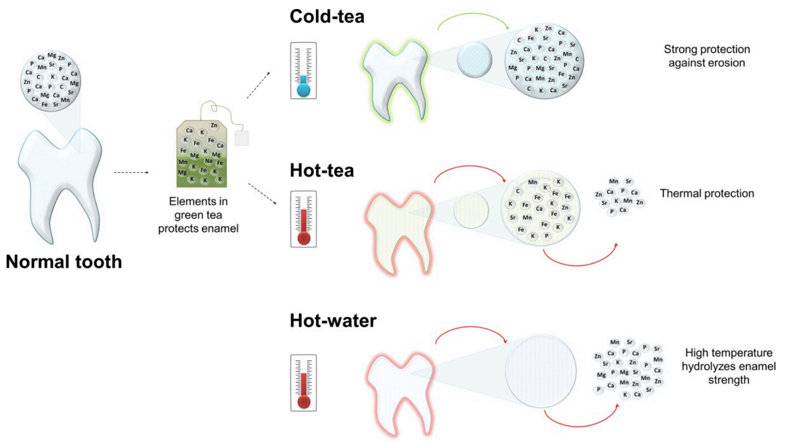

Fig 5. Schematic model illustrating the effect of tea and temperature on enamel. Healthy tooth showing a representation of the principal elements in the mineral phase (insert). There is a direct interaction between elements in teeth with the elements in tea, represented by the arrow directed toward the teabag. Cold tea favors enamel absorbing tea elements providing structural protection; although, metals pigment the enamel surface (green on the tooth). Hot tea protects against temperature although deposition of metal stains the surface. The high temperature of hot water results in the loss of elements forming the inorganic matrix.

https://doi.org/10.1371/journal.pone.0244542.g005

the hydroxyapatite structure. The HA is mainly composed of phosphate and calcium ions: $Ca_{10}(PO_4)_6 (OH)_2$, but at elevated temperatures, the structure of HA transforms to $HPO_4^{2-} + OH^- \rightarrow PO_4^{3-} + H_2O$. Because the product is water, these molecules evaporate and calcium loss occurs [51]. This abrupt damage on enamel is strongly associated with erosive demineralization.

Balance between acidity and teeth mineral elements

The pH of a solution is an important factor for demineralization [14, 69]. However, the amount of phosphate and calcium ions in the solution is also important [69]. In our study, the pH of water was >7.7, which is not considered critical for HA dissolution since previous studies indicated a pH below 5.5 dissolves enamel [70, 71]. When teeth are immersed in water, a small amount of enamel will gradually be lost because water contains minimal phosphate and calcium ions. In contrast, the pH of tea was >5.3, which could be considered critical for HA dissolution. Tea contains high concentrations of calcium and phosphates, which help to prevent enamel dissolution. Consistent with previous studies [3–7, 9, 25], our findings support the premise of tea protecting teeth against erosion.

Study limitations and future directions

The principal limitation of the present study was the short duration of the in vivo rat model (one week). Although in vivo studies of human dental tissues would be the best, it is extremely difficult to avoid confounding variables such as different dietary habits and different dental conditions (i.e. different erosive states of the dental tissue). As a result, rodents were used due to their high degree of uniform dental tissue [72] and the similarity of rat teeth to human teeth. However, we were unable to administer green tea to rodents for a long period of time due to the ancillary effects of caffeine (e.g. hyperactivity). The present experiment did not assess salivary conditions in the in vitro model. Saliva contains essential components that protect the entire oral cavity; a biofilm described as the acquired salivary pellicle. This membrane is the result of sialo- and mucoproteins derived from saliva that moisten [73], thermoregulate and protect against demineralization [74, 75]. The staining of teeth could be related to the interaction between the acquired salivary pellicle and elements which stain teeth [24]. Here, green tea might interact with both the salivary pellicle and tooth surfaces serving as a reservoir for the adherence of staining metals. Further experiments are required to identify the specific molecular mechanisms through which green tea protects enamel at cold conditions.

The present manuscript contains helpful information for future approaches in oral health. Here we suggest biomaterials to protect against high temperature could be added to dentifrices. For example, dairy products are known to reduce damage caused by aggressive beverages [75]. In this regard, adding milk to tea prevents staining due to casein and a high level of calcium and phosphate protecting the enamel against erosion [75–77]. Constructing thermoresistant dentifrices could be a new target in dental material science.

Conclusions

Here, we identified a clear pattern of elemental deposition on the enamel surface of teeth caused by tea, in addition to an increase in elements important for the conservation of teeth. As reported before, green tea can protect enamel from extrinsic damage; however, we identified a subtle erosive effect that hot temperature influences hydroxyapatite. Secondly, we identified that elements from tea interact strongly at cold conditions to protect teeth against erosion that might have a direct relation with catechins of green tea. Despite the benefits green tea possesses at cold/room temperature conditions, there is caution needed at hot temperatures. To

preserve dental structures, we recommend avoiding direct exposure to hot beverages and good hygienic habits after drinking acid/staining drinks (i.e. brushing). These preventative measures will guarantee the long-term preservation of teeth which are susceptible to erosion.

Supporting information

S1 Appendix.
(XLSX)

S2 Appendix.
(R)

S3 Appendix.
(CSV)

S4 Appendix.
(CSV)

S1 File.
(DOCX)

S2 File.
(DOCX)

Author Contributions

Conceptualization: Sinai H. C. Manno, Francis A. M. Manno, Irfan Ahmed, Vincent W. T. Li, Shisan Xu, Victor Ma, Beatriz Aldape, Shuk Han Cheng, Condon Lau.

Data curation: Li Tian, Muhammad S. Khan, Irfan Ahmed, Yuanchao Liu, Vincent W. T. Li, Fangjing Xie, Tak Fu Hung, Beatriz Aldape.

Formal analysis: Shuk Han Cheng, Condon Lau.

Funding acquisition: William Cho, Shuk Han Cheng, Condon Lau.

Investigation: Sinai H. C. Manno, William Cho, Shuk Han Cheng, Condon Lau.

Methodology: Sinai H. C. Manno.

Resources: Condon Lau.

Software: Tak Fu Hung.

Supervision: Condon Lau.

Validation: Shuk Han Cheng.

Visualization: Beatriz Aldape.

Writing – original draft: Sinai H. C. Manno, Francis A. M. Manno.

Writing – review & editing: Sinai H. C. Manno, Francis A. M. Manno.

References

1. Cheng TO. All teas are not created equal: The Chinese green tea and cardiovascular health. International Journal of Cardiology. 2006; 108(3):301–8. https://doi.org/10.1016/j.ijcard.2005.05.038 PMID: 15978686
2. Li X, Yu C, Guo Y, Bian Z, Si J, Yang L, et al. Tea consumption and risk of ischaemic heart disease. Heart. 2017; 103(10):783. https://doi.org/10.1136/heartjnl-2016-310462 PMID: 28077466

3. Kato MT, Magalhães AC, Rios D, Hannas AR, Attin T, Buzalaf MAR. Protective effect of green tea on dentin erosion and abrasion. Journal of Applied Oral Science. 2009; 17:560–4. https://doi.org/10.1590/s1678-77572009000600004 PMID: 20027426

4. Magalhães AC, Wiegand A, Rios D, Hannas A, Attin T, Buzalaf MAR. Chlorhexidine and green tea extract reduce dentin erosion and abrasion in situ. Journal of Dentistry. 2009; 37(12):994–8. https://doi.org/10.1016/j.jdent.2009.08.007 PMID: 19733206

5. Narotzki B, Reznick AZ, Aizenbud D, Levy Y. Green tea: A promising natural product in oral health. Archives of Oral Biology. 2012; 57(5):429–35. https://doi.org/10.1016/j.archoralbio.2011.11.017 PMID: 22226360

6. Passos VF, Melo MASd, Lima JPM, Marçal FF, Costa CAGdA, Rodrigues LKA, et al. Active compounds and derivatives of camellia sinensis responding to erosive attacks on dentin. Brazilian Oral Research. 2018; 32. https://doi.org/10.1590/1807-3107bor-2018.vol32.0040 PMID: 29846385

7. Hannas AR, Kato MT, Cardoso CdAB, Magalhães AC, Pereira JC, Tjäderhane L, et al. Preventive effect of toothpastes with MMP inhibitors on human dentine erosion and abrasion in vitro. Journal of Applied Oral Science. 2016; 24:61–6. https://doi.org/10.1590/1678-775720150289 PMID: 27008258

8. Maruyama T, Tomofuji T, Endo Y, Irie K, Azuma T, Ekuni D, et al. Supplementation of green tea catechins in dentifrices suppresses gingival oxidative stress and periodontal inflammation. Archives of Oral Biology. 2011; 56(1):48–53. https://doi.org/10.1016/j.archoralbio.2010.08.015 PMID: 20869695

9. Chaussain-Miller C, Fioretti F, Goldberg M, Menashi S. The Role of Matrix Metalloproteinases (MMPs) in Human Caries. Journal of Dental Research. 2006; 85(1):22–32. https://doi.org/10.1177/154405910608500104 PMID: 16373676.

10. Plate U, Arnold S, Stratmann U, Wiesmann HP, Höhling HJ. General Principle of Ordered Apatitic Crystal Formation in Enamel and Collagen Rich Hard Tissues. Connective Tissue Research. 1998; 38(1–4):149–57. https://doi.org/10.3109/03008209809017032 PMID: 11063023

11. Moradian-Oldak J. Protein-mediated enamel mineralization. Front Biosci (Landmark Ed). 2012; 17:1996–2023. Epub 2012/06/02. https://doi.org/10.2741/4034 PMID: 22652761; PubMed Central PMCID: PMC3442115.

12. Dusevich V, Melander JR, Eick JD. SEM in dental research. In: Schatten H, editor. Scanning Electron Microscopy for the Life Sciences. Advances in Microscopy and Microanalysis. Cambridge: Cambridge University Press; 2012. p. 211–35.

13. Ganss C. Definition of Erosion and Links to Tooth Wear: Basel, New York, Karger; 2006.

14. Larsen MJ. Dissolution of enamel. Scand J Dent Res. 1973; 81(7):518–22. Epub 1973/01/01. https://doi.org/10.1111/j.1600-0722.1973.tb00358.x PMID: 4520615.

15. Barbour ME, Finke M, Parker DM, Hughes JA, Allen GC, Addy M. The relationship between enamel softening and erosion caused by soft drinks at a range of temperatures. Journal of Dentistry. 2006; 34(3):207–13. https://doi.org/10.1016/j.jdent.2005.06.002 PMID: 16112333

16. Amaechi BT, Higham SM, Edgar WM. Use of transverse microradiography to quantify mineral loss by erosion in bovine enamel. Caries Res. 1998; 32(5):351–6. Epub 1998/08/14. https://doi.org/10.1159/000016471 PMID: 9701660.

17. O'Toole S, Mullan F. The role of the diet in tooth wear. British Dental Journal. 2018; 224(5):379–83. https://doi.org/10.1038/sj.bdj.2018.127 PMID: 29471309

18. Nunn J. Influence of diet on dental erosion in 14-year-olds. British Dental Journal. 2001; 190(5):250–. https://doi.org/10.1038/sj.bdj.4800939

19. Lussi A, Megert B, Peter Shellis R, Wang X. Analysis of the erosive effect of different dietary substances and medications. British Journal of Nutrition. 2012; 107(2):252–62. Epub 2011/06/30. https://doi.org/10.1017/S0007114511002820 PMID: 21733310

20. Bartlett DW, Fares J, Shirodaria S, Chiu K, Ahmad N, Sherriff M. The association of tooth wear, diet and dietary habits in adults aged 18–30 years old. Journal of Dentistry. 2011; 39(12):811–6. https://doi.org/10.1016/j.jdent.2011.08.014 PMID: 21911033

21. Graham DY, Abou-Sleiman J, El-Zimaity HMT, Badr A, Graham DP, Malaty HM. Helicobacter pylori Infection, Gastritis, and the Temperature of Choice for Hot Drinks. Helicobacter. 1996; 1(3):172–4. https://doi.org/10.1111/j.1523-5378.1996.tb00034.x PMID: 9398901

22. Brown F, Diller KR. Calculating the optimum temperature for serving hot beverages. Burns. 2008; 34(5):648–54. https://doi.org/10.1016/j.burns.2007.09.012 PMID: 18226454

23. West NX, Hughes JA, Addy M. Erosion of dentine and enamel in vitro by dietary acids: the effect of temperature, acid character, concentration and exposure time. Journal of Oral Rehabilitation. 2000; 27(10):875–80. https://doi.org/10.1046/j.1365-2842.2000.00583.x PMID: 11065022

24. Simpson A, Shaw L, Smith AJ. Tooth surface pH during drinking of black tea. British Dental Journal. 2001; 190(7):374–6. https://doi.org/10.1038/sj.bdj.4800977 PMID: 11338040

25. Ozan G, Sar Sancakli H, Yucel T. Effect of black tea and matrix metalloproteinase inhibitors on eroded dentin in situ. Microscopy Research and Technique. 2020; 83(7):834–42. https://doi.org/10.1002/jemt.23475 PMID: 32196821

26. Manno SHC, Manno FAM, Ahmed I, Ahmed R, Shu L, Li L, et al. Spectroscopic examination of enamel staining by coffee indicates dentin erosion by sequestration of elements. Talanta. 2018; 189:550–9. https://doi.org/10.1016/j.talanta.2018.07.032 PMID: 30086959

27. Hahn DW, Omenetto N. Laser-Induced Breakdown Spectroscopy (LIBS), Part II: Review of Instrumental and Methodological Approaches to Material Analysis and Applications to Different Fields. Applied Spectroscopy. 2012; 66(4):347–419. https://doi.org/10.1366/11-06574 PMID: 22449322.

28. Ahmed I, Manno FAM, Manno SHC, Liu Y, Zhang Y, Lau C. Detection of lithium in breast milk and in situ elemental analysis of the mammary gland. Biomed Opt Express. 2018; 9(9):4184–95. https://doi.org/10.1364/BOE.9.004184 PMID: 30615726

29. Ahmed I, Yang J, Law AWL, Manno FAM, Ahmed R, Zhang Y, et al. Rapid and in situ optical detection of trace lithium in tissues. Biomed Opt Express. 2018; 9(9):4459–71. https://doi.org/10.1364/BOE.9.004459 PMID: 30615723

30. Ahmed I, Ahmed R, Yang J, Law AWL, Zhang Y, Lau C. Elemental analysis of the thyroid by laser induced breakdown spectroscopy. Biomed Opt Express. 2017; 8(11):4865–71. https://doi.org/10.1364/BOE.8.004865 PMID: 29188087

31. Yueh F-Y, Zheng H, Singh JP, Burgess S. Preliminary evaluation of laser-induced breakdown spectroscopy for tissue classification. Spectrochimica Acta Part B: Atomic Spectroscopy. 2009; 64(10):1059–67.

32. Imam H, Ahmed D, Eldakrouri A. Elemental content of enamel and dentin after bleaching of teeth (a comparative study between laser-induced breakdown spectroscopy and x-ray photoelectron spectroscopy). Journal of Applied Physics. 2013; 113(23):234701. https://doi.org/10.1063/1.4811186

33. Samek O, Beddows D, Telle H, Morris G, Liska M, Kaiser J. Quantitative analysis of trace metal accumulation in teeth using laser-induced breakdown spectroscopy. Applied Physics A. 1999; 69(1):S179–S82.

34. Samek O, Telle HH, Beddows DCS. Laser-induced breakdown spectroscopy: a tool for real-time, in vitro and in vivo identification of carious teeth. BMC Oral Health. 2001; 1(1):1. https://doi.org/10.1186/1472-6831-1-1 PMID: 11801201

35. Gazmeh M, Bahreini M, Tavassoli SH. Discrimination of healthy and carious teeth using laser-induced breakdown spectroscopy and partial least square discriminant analysis. Appl Opt. 2015; 54(1):123–31. https://doi.org/10.1364/AO.54.000123 PMID: 25967014

36. Liu Y, Hsieh Y-K, Chu Y, Ahmed I, Hu Z, Khan MS, et al. Quantitative trace elemental analysis by laser-induced breakdown spectroscopy with dried droplet pretreatment. Journal of Analytical Atomic Spectrometry. 2020. https://doi.org/10.1039/c9ja00344d PMID: 32669750

37. Poggio C, Lombardini M, Colombo M, Bianchi S. Impact of two toothpastes on repairing enamel erosion produced by a soft drink: An AFM in vitro study. Journal of Dentistry. 2010; 38(11):868–74. https://doi.org/10.1016/j.jdent.2010.07.010 PMID: 20673785

38. Poggio C, Lombardini M, Vigorelli P, Ceci M. Analysis of dentin/enamel remineralization by a CPP-ACP paste: AFM and SEM study. Scanning. 2013; 35(6):366–74. https://doi.org/10.1002/sca.21077 PMID: 23427062

39. Farrokhpay S. Application of Spectroscopy and Microscopy Techniques in Surface Coatings Evaluation: A Review. Applied Spectroscopy Reviews. 2012; 47(3):233–43. https://doi.org/10.1080/05704928.2011.639424

40. Lussi A, Bossen A, Hoschele C, Beyeler B, Megert B, Meier C, et al. Effects of enamel abrasion, salivary pellicle, and measurement angle on the optical assessment of dental erosion. Journal of Biomedical Optics. 2012; 17(9):097009.

41. Jälevik B, Dietz W, Norén JG. Scanning electron micrograph analysis of hypomineralized enamel in permanent first molars. International Journal of Paediatric Dentistry. 2005; 15(4):233–40. https://doi.org/10.1111/j.1365-263X.2005.00644.x PMID: 16011781

42. Dinçer B, Hazar S, Sen BH. Scanning electron microscope study of the effects of soft drinks on etched and sealed enamel. American Journal of Orthodontics and Dentofacial Orthopedics. 2002; 122(2):135–41. https://doi.org/10.1067/mod.2002.124458 PMID: 12165767

43. Rugg-Gunn AJ, Maguire A, Gordon PH, McCabe JF, Stephenson G. Comparison of Erosion of Dental Enamel by Four Drinks Using an Intra-Oral Applicance. Caries Research. 1998; 32(5):337–43. https://doi.org/10.1159/000016469 PMID: 9701658

44. 14084 BSDBE. Foodstuffs. Determination of trace elements. Determination of lead, cadmium, zinc, copper and iron by atomic absorption spectrometry (AAS) after microwave digestion. https://doi.org/10.3403/02794284u

45. Alvira F, Ramirez Rozzi F, Torchia G, Roso L, Bilmes G. A new method for relative Sr determination in human teeth enamel. J Anthropol Sci. 2011; 89:153–60. Epub 2011/07/16. https://doi.org/10.4436/jass.89012 PMID: 21757792.

46. Alvira FC, Ramirez Rozzi F, Bilmes GM. Laser-Induced Breakdown Spectroscopy Microanalysis of Trace Elements in Homo sapiens Teeth. Applied Spectroscopy. 2010; 64(3):313–9. https://doi.org/10.1366/000370210790918328 PMID: 20223067

47. Hamzaoui S, Nouir R, Jaidene N. The Study of Carious Teeth by Laser-Induced Breakdown Spectroscopy. Journal of Applied Spectroscopy. 2017; 84(1):82–6. https://doi.org/10.1007/s10812-017-0431-5

48. Matsuura Y, editor Detection of Early Caries by Laser-Induced Breakdown Spectroscopy. Interface Oral Health Science 2016; 2017 2017; Singapore: Springer Singapore.

49. Singh VK, Rai AK. Potential of laser-induced breakdown spectroscopy for the rapid identification of carious teeth. Lasers Med Sci. 2011; 26(3):307–15. Epub 2010/04/24. https://doi.org/10.1007/s10103-010-0786-9 PMID: 20414707.

50. Annabi B, Lachambre M-P, Bousquet-Gagnon N, Pagé M, Gingras D, Béliveau R. Green tea polyphenol (−)-epigallocatechin 3-gallate inhibits MMP-2 secretion and MT1-MMP-driven migration in glioblastoma cells. Biochimica et Biophysica Acta (BBA)—Molecular Cell Research. 2002; 1542(1):209–20. https://doi.org/10.1016/s0167-4889(01)00187-2 PMID: 11853893

51. Shpak A, Karbovskii V, Vakhney A, Kluyenko L. Spectral and quantum-mechanical study of electronic structure of ultradispersed calcium hydroxyapatite. Surface. 2002;(7–8):57–68.

52. Speirs RL. The nature of surface enamel in human teeth. Calcified Tissue Research. 1971; 8(1):1–16. https://doi.org/10.1007/BF02010118 PMID: 4944366

53. Prskalo K. Risk Factors Associated with Black Tooth Stain. Acta Clin Croat. 2017; 56:28–35. https://doi.org/10.20471/acc.2017.56.01.05 PMID: 29120125

54. Rebelo de Sousa K, Batista MJ, Rocha Gonçalves J, Da Luz Rosário de Sousa M. Extrinsic Tooth Enamel Color Changes and Their Relationship with the Quality of Water Consumed. International Journal of Environmental Research and Public Health. 2012; 9(10):3530–9. https://doi.org/10.3390/ijerph9103530 PMID: 23202761

55. Enomoto A, Tanaka T, Kawagishi S, Nakashima H, Watanabe K, Maki K. Amounts of Sr and Ca eluted from deciduous enamel to artificial saliva related to dental caries. Biol Trace Elem Res. 2012; 148 (2):170–7. Epub 2012/03/07. https://doi.org/10.1007/s12011-012-9368-y PMID: 22391794.

56. Pan HB, Li ZY, Lam WM, Wong JC, Darvell BW, Luk KDK, et al. Solubility of strontium-substituted apatite by solid titration. Acta Biomaterialia. 2009; 5(5):1678–85. https://doi.org/10.1016/j.actbio.2008.11.032 PMID: 19135423

57. Wang Y-L, Chang H-H, Chiang Y-C, Lin C-H, Lin C-P. Strontium ion can significantly decrease enamel demineralization and prevent the enamel surface hardness loss in acidic environment. Journal of the Formosan Medical Association. 2019; 118(1, Part 1):39–49. https://doi.org/10.1016/j.jfma.2018.01.001 PMID: 29395390

58. Humphrey LT, Dean MC, Jeffries TE, Penn M. Unlocking evidence of early diet from tooth enamel. Proceedings of the National Academy of Sciences. 2008; 105(19):6834. https://doi.org/10.1073/pnas.0711513105 PMID: 18458343

59. Mullan F, Austin RS, Parkinson CR, Hasan A, Bartlett DW. Measurement of surface roughness changes of unpolished and polished enamel following erosion. PLOS ONE. 2017; 12(8):e0182406. https://doi.org/10.1371/journal.pone.0182406 PMID: 28771562

60. Lechner B-D, Röper S, Messerschmidt J, Blume A, Magerle R. Monitoring Demineralization and Subsequent Remineralization of Human Teeth at the Dentin–Enamel Junction with Atomic Force Microscopy. ACS Applied Materials & Interfaces. 2015; 7(34):18937–43. https://doi.org/10.1021/acsami.5b04790 PMID: 26266571

61. Quartarone E, Mustarelli P, Poggio C, Lombardini M. Surface kinetic roughening caused by dental erosion: An atomic force microscopy study. Journal of Applied Physics. 2008; 103(10):104702. https://doi.org/10.1063/1.2927386

62. Sa Y, Chen D, Liu Y, Wen W, Xu M, Jiang T, et al. Effects of two in-office bleaching agents with different pH values on enamel surface structure and color: an in situ vs. in vitro study. J Dent. 2012; 40 Suppl 1: e26–34. https://doi.org/10.1016/j.jdent.2012.02.010 PMID: 22381613.

63. Sato C, Rodrigues FA, Garcia DM, Vidal CMP, Pashley DH, Tjäderhane L, et al. Tooth bleaching increases dentinal protease activity. Journal of dental research. 2013; 92(2):187–92. Epub 2012/12/14. https://doi.org/10.1177/0022034512470831 PMID: 23242228.

64. Sun L, Liang S, Sa Y, Wang Z, Ma X, Jiang T, et al. Surface alteration of human tooth enamel subjected to acidic and neutral 30% hydrogen peroxide. J Dent. 2011; 39(10):686–92. Epub 2011/08/23. https://doi.org/10.1016/j.jdent.2011.07.011 PMID: 21855600.

65. Quirynen M, Bollen CM. The influence of surface roughness and surface-free energy on supra- and subgingival plaque formation in man. A review of the literature. J Clin Periodontol. 1995; 22(1):1–14. Epub 1995/01/01. https://doi.org/10.1111/j.1600-051x.1995.tb01765.x PMID: 7706534.

66. Quirynen M, Marechal M, Busscher HJ, Weerkamp AH, Darius PL, van Steenberghe D. The influence of surface free energy and surface roughness on early plaque formation. An in vivo study in man. J Clin Periodontol. 1990; 17(3):138–44. Epub 1990/03/01. https://doi.org/10.1111/j.1600-051x.1990.tb01077.x PMID: 2319000.

67. Rashid H. The effect of surface roughness on ceramics used in dentistry: A review of literature. Eur J Dent. 2014; 8(4):571–9. https://doi.org/10.4103/1305-7456.143646 PMID: 25512743.

68. Hemingway CA, Parker DM, Addy M, Barbour ME. Erosion of enamel by non-carbonated soft drinks with and without toothbrushing abrasion. British Dental Journal. 2006; 201(7):447–50. https://doi.org/10.1038/sj.bdj.4814073 PMID: 17031352

69. Dawes C. What is the critical pH and why does a tooth dissolve in acid? J Can Dent Assoc. 2003; 69 (11):722–4. Epub 2003/12/05. PMID: 14653937.

70. Alavi G, Alavi A, Saberfiroozi M, Sarbazi A, Motamedi M, Hamedani S. Dental Erosion in Patients with Gastroesophageal Reflux Disease (GERD) in a Sample of Patients Referred to the Motahari Clinic, Shiraz, Iran. J Dent (Shiraz). 2014; 15(1):33–8. PMID: 24738088.

71. Barron RP, Carmichael RP, Marcon MA, Sàndor GK. Dental erosion in gastroesophageal reflux disease. J Can Dent Assoc. 2003; 69(2):84–9. PMID: 12559056.

72. Goldberg M, Kellermann O, Dimitrova-Nakov S, Harichane Y, Baudry A. Comparative studies between mice molars and incisors are required to draw an overview of enamel structural complexity. Frontiers in Physiology. 2014; 5(359). https://doi.org/10.3389/fphys.2014.00359 PMID: 25285079

73. K AJ. Oral development and histology Third ed: Thieme Medical Publishers; 2002. 435 p.

74. Amerongen AVN, Oderkerk CH, Driessen AA. Role of Mucins from Human Whole Saliva in the Protection of Tooth Enamel against Demineralization in vitro. Caries Research. 1987; 21(4):297–309. https://doi.org/10.1159/000261033 PMID: 3475175

75. Gedalia I, Ionat-Bendat D, Ben-Mosheh S, Shapira L. Tooth enamel softening with a cola type drink and rehardening with hard cheese or stimulated saliva in situ. Journal of Oral Rehabilitation. 1991; 18 (6):501–6. https://doi.org/10.1111/j.1365-2842.1991.tb00072.x PMID: 1762023

76. Lee RJ, Bayne A, Tiangco M, Garen G, Chow AK. Prevention of tea-induced extrinsic tooth stain. International Journal of Dental Hygiene. 2014; 12(4):267–72. https://doi.org/10.1111/idh.12096 PMID: 25040739

77. Battistella E, Mele S, Rimondini L. Dental tissue engineering: a new approach to dental tissue reconstruction. Biomimetics learning from nature. 2010 Mar 1.
